# FDG uptake correlates with recurrence and survival after treatment of unresectable stage III non-small cell lung cancer with high-dose proton therapy and chemotherapy

**DOI:** 10.1186/1748-717X-7-144

**Published:** 2012-08-28

**Authors:** Zuo-Lin Xiang, Jeremy Erasmus, Ritsuko Komaki, James D Cox, Joe Y Chang

**Affiliations:** 1 Departments of Radiation Oncology, The University of Texas MD Anderson Cancer Center, 1515 Holcombe Blvd., Houston, TX, 77030, USA; 2 Departments of Diagnostic Radiology, The University of Texas MD Anderson Cancer Center, Houston, TX, USA

**Keywords:** Proton therapy, Chemotherapy, Non-small cell lung cancer, PET/CT imaging, Standardized uptake value, Prognostic factors

## Abstract

**Background:**

We studied whether maximum standardized uptake values (SUV) from [^18^ F] PET/CT predict clinical outcome after concurrent proton/chemotherapy for stage III non-small cell lung cancer (NSCLC).

**Methods:**

Eighty-four patients were treated prospectively with 74 Gy(RBE) proton therapy and concurrent chemotherapy. PET/CT scans were available before (SUV1) and within 6 months after (SUV2) treatment. The predictive value of clinical and PET/CT factors were analyzed with univariate and multivariate Cox regression models.

**Results:**

Median survival time was 29.9 months. At 3 years, the local recurrence-free survival (LRFS) rate was 34.8%; distant metastasis-free survival (DMFS), 35.4%; progression-free survival (PFS), 31.2%; and overall survival (OS), 37.2%. Patients with SUV2 ≥3.6 (the median) had high rates of LR (*p* = 0.021). Of 12 clinicopathologic features evaluated in univariate analysis, only KPS, SUV1, and SUV2 predicted LRFS, DMFS, PFS, and OS (*p* <0.05). Multivariate analysis showed that KPS (*p* = 0.025) and SUV2 (*p* = 0.017) were independently prognostic for LRFS and that SUV1, SUV2, and KPS were independently prognostic for DMFS, PFS, and OS (*p* <0.05).

**Conclusions:**

SUV2 predicted LRFS, and SUV1 and SUV2 predicted DMFS, PFS, and OS, in patients with stage III NSCLC treated with concurrent chemotherapy and high-dose proton therapy.

## Background

Non-small cell lung cancer (NSCLC) is the leading cause of cancer death worldwide. About one-third of patients with NSCLC present with locally advanced disease, the standard treatment for which is concurrent chemoradiation [1]. However, conventional doses of 60 Gy of photon (x-ray) radiation produce local failure rates of about 50%, and dose escalation has been associated with increased toxicity, particularly when chemotherapy is given concurrently with the radiation [1,2]. For some patients, proton therapy may provide better dose distributions than photon therapy in that protons allow delivery of similar or higher doses to malignant tissues while delivering less radiation to normal tissues, which could allow safe dose escalation [3,4]. Early results of a phase II study of high-dose proton therapy involving doses of 74 Gy(RBE) (assuming a relative biological effectiveness [RBE] factor of 1.1) given with concurrent weekly carboplatin and paclitaxel to 44 patients with inoperable stage III NSCLC indicated favorable toxicity and promising overall survival compared with published findings [5]. However, about 20% of these patients experienced local recurrence and 40% distant recurrence. Implementation of individualized therapy would be greatly facilitated by ways of accurately assessing the extent of disease, both before treatment (staging) and afterward (response to therapy). One modality being studied intensively for this purpose is ^18^ F-fluorodeoxyglucose positron emission tomography (^18^ F-FDG PET), with or without integrated computed tomography (CT). ^18^ F-FDG PET has been shown to improve the rate of detection of regional and distant metastases in patients with NSCLC [6], and ^18^ F-FDG PET/CT was found to be more accurate in staging NSCLC than visual correlation of separate PET and CT images [7]. PET/CT is also highly accurate for detecting tumor growth and disease progression in NSCLC after therapy [8]. PET/CT imaging can also be useful for delineating radiotherapy target volumes so that elective nodal irradiation can be avoided but the dose to PET-avid lesions escalated [9,10]. PET/CT has been used to predict response to chemotherapy and to predict clinical outcome in stage III NSCLC treated with conventional (photon-based) radiotherapy to 60–63 Gy [11-13]. However, false-positive findings from therapy-induced inflammation can be a problem for PET scans obtained after therapy [14] particularly when higher doses are used.

To date, the potential predictive value of PET/CT for stage III NSCLC treated with high-dose [74 Gy(RBE)] radiotherapy combined with concurrent chemotherapy has not been reported. To address this gap, we sought to determine whether maximum standardized uptake values (SUV max) from ^18^ F-FDG PET/CT and other clinicopathologic features could predict recurrence and survival in such patients.

## Methods

### Study population

All patients included in this analysis had been enrolled in one of two phase II prospective studies of proton therapy (clinicaltrials.gov identifiers NCT00495170 and NCT00991094); both protocols included as a secondary objective determining the predictive value of PET/CT in clinical outcomes, and both had been approved by the institutional review board of MD Anderson Cancer Center. For this analysis, 84 patients with unresectable confirmed stage III NSCLC were retrospectively identified based on the availability of pretreatment and posttreatment PET/CT images and SUVs. Patients without complete PET/CT images and SUVs or those with images of poor image quality were excluded. Disease in all cases had been staged with magnetic resonance imaging or CT of the brain, CT of the chest, and PET/CT.

### Treatment simulation and target volume delineation

Treatment was simulated for all patients with 4-dimensional (4D) CT to account for tumor motion. The internal gross tumor volume (iGTV) was defined as the envelope of motion of the GTV on a reconstructed maximum intensity projection image and was verified across all phases of the 4D CT dataset [15]. Staging PET/CT images were fused with 4D CT treatment simulation images to delineate the gross tumor if the patient’s position was the same for the PET/CT and treatment simulation procedures. Otherwise, PET images were used as a guide to help delineate the GTV. The primary tumor and clinically positive lymph nodes seen either on the planning CT scan (>1 cm short axis diameter) or on the pretreatment PET scan (SUV >3) constituted the GTV. Any lymph node suspected of harboring disease on clinical evaluation that was negative on imaging was subjected to endobranchial sonography or medianoscopy-based biopsy. An 8-mm isotropic expansion of the iGTV was added and edited to cover possible microextension of the tumor or lymph nodes adjacent to gross tumor, and the resulting volume was defined as the internal clinical target volume. Uninvolved lymph nodes were not irradiated intentionally.

### Radiation doses

The total radiation dose to the tumor target was 74 Gy(RBE), given in once-daily 2-Gy(RBE) fractions, 5 days per week, over 7 weeks. Treatment plans were designed in accordance with previously described dose-volume constraints [5].

### Passive scattering proton therapy planning and adaptive proton delivery

The iGTV, with the maximum intensity projection density from the set of 3-dimensional CT scans used to derive the 4D CT, was used to design compensators and apertures to account for tumor motion, and the treatment plan was calculated by using the average of the phases of the 4D CT [3,15-17]. Another set of 4D CT scans was obtained during week 3 or 4 of treatment (or as clinically indicated as assessed by the treating physician) to document tumor shrinkage or other anatomic or motion-based changes. If the new dose distribution derived from these changes could not meet the minimum target dose requirement of ≥95% of the prescribed dose, or if it exceeded normal tissue dose constraints, a new treatment plan was designed for the remainder of the treatments.

### Chemotherapy

All patients received concurrent carboplatin (2 AUC) and paclitaxel (50 mg/m^2^) as weekly intravenous infusions during proton therapy. Neoadjuvant chemotherapy and adjuvant chemotherapy with carboplatin and paclitaxel at systemic doses were allowed.

### Evaluation and follow-up

Patients were evaluated weekly during treatment, at 6 weeks after completing proton therapy, every 3 months for 2 years, and every 6 months thereafter. The follow-up visit included interval medical history and physical examination, hematologic studies, and CT. Follow-up PET/CT was required during the first 1–6 months after treatment and afterward as needed.

Local control at the primary tumor site was evaluated by serial thoracic CT scans with contrast. If CT scans showed evidence of recurrent disease, PET or PET/CT was required and biopsy recommended to confirm recurrence. Particular attention was paid to patterns of FDG uptake with respect to radiation field and tumor location to avoid confusing inflammation from recurrence; FDG uptake coincident with the radiation fields extending beyond the primary tumor boundaries was considered pneumonitis, not tumor recurrence. All images were reviewed by diagnostic radiologists and radiation oncologists specializing in thoracic disease. Unconfirmed recurrent disease was to be followed up with CT or PET. The timing of the recurrence was scored as the time at which the first image (PET and/or CT) showed abnormalities. Local recurrence was defined as recurrent disease within the planning target volume (PTV). Nodal recurrence outside this volume but within the chest or in the supraclavicular area was documented separately as regional recurrence.

### PET/CT scans

All patients received a median 499.5 MBq (range, 277.5–740 MBq) intravenous injection of ^18^ F-FDG before imaging, followed by a 60-minute uptake phase. All scans were obtained with a GE Discovery ST PET/CT scanner, with vendor-provided software used to interpret image data. Scans were all acquired in two dimensions at 5 minutes per field of view, and non-contrast CT images were used for attenuation correction. Patients were to have fasted for at least 6 hours before the PET scan and to have a blood glucose level of <150 mg/dL at the time of injection. Regions of interest were manually drawn on the transaxial images around the focal ^18^ F-FDG uptake zone in the primary tumor, and the SUVmax for each patient was used to minimize partial-volume effects. ^18^ F-FDG PET/CT scans were obtained from all patients before chemoradiotherapy (SUV1, ≤3 months) and after chemoradiotherapy (SUV2, ≤6 months); delta (Δ) SUV was SUV1 minus SUV2.

### Statistical analysis

Statistical analyses were done with SPSS 16.0 software (SPSS, Chicago, IL). Medians were used as cutoff values. Start dates for all survival estimates were the date of protocol enrollment. Local recurrence–free survival (LRFS) was defined as the interval from that date to the date of LR or death; distant metastasis–free survival (DMFS) from that date to the date of DM or death; progression-free survival (PFS) from that date to the date of tumor recurrence, DM, progressive disease, or death; and overall survival (OS) from that date to the date of death. Cumulative LRFS, DMFS, PFS, and OS rates were estimated using the Kaplan-Meier method, and groups of interest were compared by using log-rank tests. Univariate and multivariate survival analyses were based on the Cox proportional hazards regression model. A two-tailed *p* <0.05 indicated statistical significance.

## Results

### Characteristics of patients and clinical outcomes

**Figure 1:**
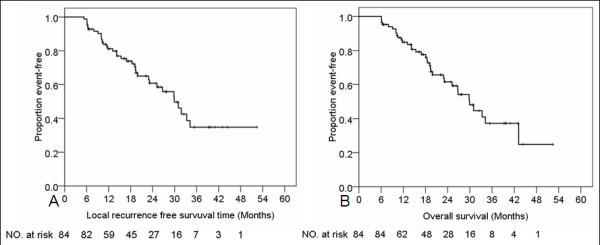
Local-regional recurrence–free survival (A) and overall survival (B) curves for all 84 patients.

Patient characteristics, including SUVs before and after treatment, are shown in Table 1. For SUV1, the median time from PET/CT scan to radiotherapy was 0.7 month (range 0.1–2.7 months), and median SUV1 was 14.2 (range 2.5–66). For SUV2, the median time from radiotherapy to follow-up PET/CT scan was 4.2 months (range 0.8-6.0 months), and median SUV2 was 3.6 (range 1.9–28.1). Median follow-up time for all patients was 19.2 months (range, 6.1–52.4 months). All patients received 74 Gy(RBE) proton therapy and concurrent weekly chemotherapy, and 22 (26%) also received induction chemotherapy. Fourteen patients (17%) had LR, but only 7 (8%) had isolated LR (i.e., LR without RR or DM). The 2-year local control rate was 83.3%. Three patients (4%) had regional lymph node recurrence inside the PTV (i.e., LR). Seven patients (8%) had regional lymph node recurrence (RR) outside the PTV, but only 2 (2%) had isolated lymph node recurrence (i.e., RR without LR or DM). Thirty-three patients (39%) had DM, and 36 patients (43%) had died at the last follow-up. Median survival time for all patients was 29.9 months. Survival rates at 3 years were 34.8% (LRFS), 35.4% (DMFS), 31.2% (PFS), and 37.2% (OS). LRFS and OS rates are shown in Figure 1.

**Table 1:** Clinicopathologic characteristics of 84 unresectable stage III non-small-cell lung cancer patients

**Characteristic**	**Patients**
Age (y)	
Median, year (range)	70 (37–87)
Gender	
Male	55 (65.5%)
Female	29 (34.5%)
KPS	
Median (range)	90 (70–100)
Smoking history	
Yes	77 (91.7%)
No	7 (8.3%)
Histology	
Squamous	38 (45.2%)
Adenocarcinoma	34 (40.5%)
NSC NOS	12 (14.3%)
GTV	
Median, cm^3^ (range)	96.6 (4.1-753.2)
SUV1	
Median value (range)	14.2 (2.5-66)
Median time, month (range)	0.7 (0.1-2.7)
SUV2	
Median value (range)	3.6 (1.9-28.1)
Median time, month (range)	4.2 (0.8-6.0)

*Abbreviations:* KPS = Karnofsky Performance Status; NSC NOS = non-small cell cancer and no otherwise specified; GTV = gross tumor volume; SUV = standardized uptake values.

### SUV as a prognostic factor

**Figure 2:**
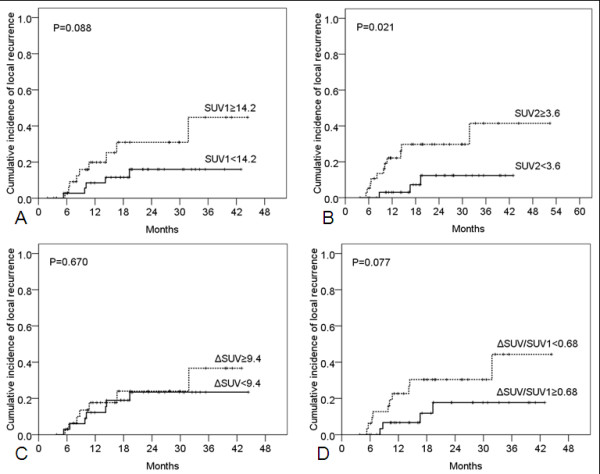
**Kaplan-Meier and log-rank analyses of cumulative local recurrence rates according to standardized uptake values obtained before therapy (SUV1; A), after therapy (SUV2; B), the difference between SUV1 and SUV2 (ΔSUV; C), and ΔSUV/SUV1 (D).** Only SUV2 was significantly associated with local recurrence.

For all 84 patients, censored time-to-event data for LR and DM were analyzed with the Kaplan-Meier method and compared with log-rank tests. Patients with SUV2 ≥3.6 were found to have higher LR rates (*p* = 0.021; Figure 2). Neither SUV1 (*p* = 0.088) nor ΔSUV (*p* = 0.670) nor ΔSUV/SUV1 (*p* = 0.077), all dichotomized at the median, correlated with LR. Patients with SUV1 ≥14.2 (*p* = 0.012) and SUV2 ≥3.6 (*p* = 0.010) had higher rates of DM, but neither ΔSUV (*p* = 0.104) nor ΔSUV/SUV1 (*p* = 0.921) were associated with DM.

**Figure 3:**
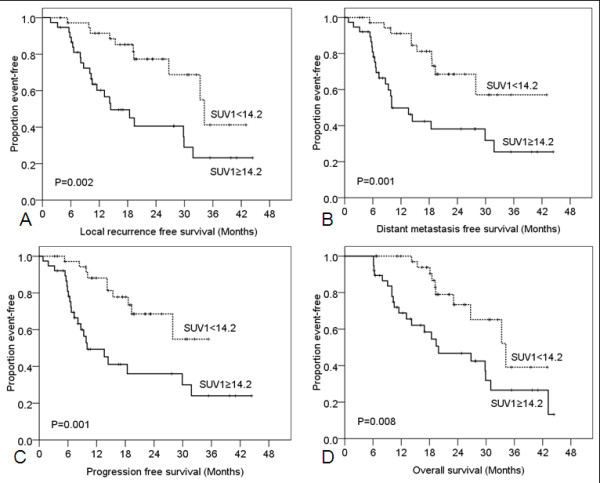
**Kaplan-Meier analysis of survival rates according to the standardized uptake value obtained before therapy (SUV1).** Patients with SUV1 ≥14.2 worse lower local recurrence–free survival (**A**), distant metastasis–free survival (**B**), progression-free survival (**C**), and overall survival (**D**) relative to those with SUV1 <14.2.

**Figure 4:**
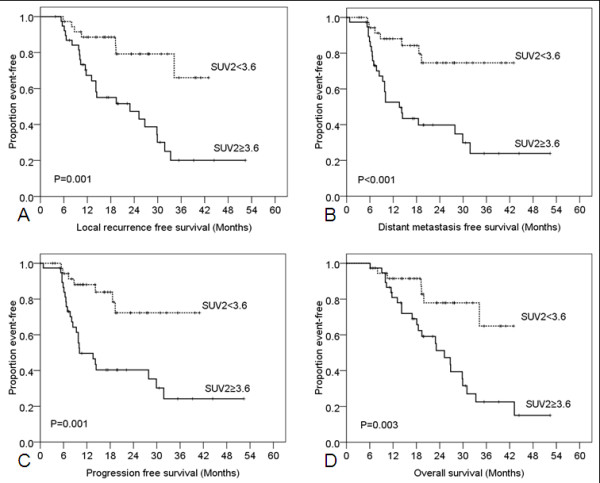
**Kaplan-Meier analysis of survival rates according to the standardized uptake value obtained after therapy (SUV2).** Patients with SUV2 ≥3.6 had worse local recurrence–free survival (**A**), distant metastasis–free survival (**B**), progression-free survival (**C**), and overall survival (**D**) relative to those with SUV2 <3.6.

Patients with SUV1 ≥14.2 had worse LRFS, DMFS, PFS, and OS (Figure 3). Patients with SUV2 ≥3.6 had poorer LRFS, DMFS, PFS, and OS (Figure 4) than those with SUV2 < 3.6. ΔSUV (dichotomized at the median) was not related to LRFS (*p* = 0.405), DMFS (*p* = 0.229), PFS (*p* = 0.151), or OS (*p* = 0.411). Likewise, ΔSUV/SUV1 (dichotomized at the median) was also not related to LRFS (*p* = 0.260), DMFS (*p* = 0.375), PFS (*p* = 0.486), or OS (*p* = 0.579).

Twelve clinicopathologic features were considered in the Cox proportional hazards regression univariate analysis: age, sex, Karnofsky performance status (KPS), smoking status, tumor histology, GTV, lung V_20_ , mean lung dose, SUV1, SUV2, ΔSUV, and ΔSUV/SUV1. Univariate analysis showed that KPS, SUV1, and SUV2 were associated with LRFS, DMFS, PFS, and OS (Table 2). Multivariate Cox proportional hazards regression analyses revealed that KPS (*p* = 0.025) and SUV2 (*p* = 0.017) were independent prognostic factors for LRFS; KPS, SUV1, and SUV2 were independent prognostic factors for DMFS, PFS, and OS (Table 2).

**Table 2:** Univariate and multivariate analyses of factors associated with survival in a cohort of 84 unresectable stage III non-small-cell lung cancer patients

**Variable**	**LRFS**		**DMFS**		**PFS**		**OS**	
	**HR 95% CI**	**p**	**HR 95% CI**	**p**	**HR 95%CI**	**p**	**HR 95%CI**	**p**
Univariate analyses								
Age (< 70 vs ≥ 70 years)	0.837,0.425-1.652	0.609	0.820,0.417-1.615	0.566	0.792,0.402-1.560	0.500	0.902,0.456-1.783	0.766
Gender (female vs male)	1.238,0.606-2.532	0.558	1.283,0.628-2.623	0.495	1.253,0.612-2.564	0.538	1.107,0.538-2.275	0.782
KPS (< 90 vs ≥ 90)	0.479,0.245-0.938	0.032	0.496,0.253-0.971	0.041	0.509,0.260-0.996	0.049	0.424,0.216-0.831	0.012
Smoking history(Yes vs No)	0.249,0.034-1.822	0.171	0.236,0.032-1.726	0.155	0.260,0.036-1.902	0.185	0.249,0.034-1.822	0.171
Histology (SCCvs Adeno)	0.593,0.287-1.223	0.157	0.556,0.270-1.147	0.112	0.538,0.261-1.111	0.094	0.637,0.309-1.313	0.222
GTV, cm^3^ (<96.6 vs ≥ 96.6)	1.664,0.817-3.309	0.163	1.718,0.855-3.453	0.129	1.734,0.863-3.483	0.122	1.764,0.866-3.592	0.118
Lung V20 (26.2 < vs ≥26.2)	1.057,0.544-2.053	0.871	0.967,0.497-1.881	0.921	1.002,0.515-1.947	0.996	1.005,0.517-1.955	0.989
MLD (1797.1 < vs ≥1797.1)	1.489,0.744-2.979	0.260	1.522,0.767-3.018	0.229	1.528,0.771-3.031	0.225	1.489,0.744-2.979	0.260
SUV1 (< 14.2 vs ≥ 14.2)	3.029,1.426-6.432	0.004	3.204,1.504-6.827	0.003	3.269,1.529-6.992	0.002	2.670,1.251-5.699	0.011
SUV2 (< 3.6 vs ≥ 3.6)	3.823,1.646-8.881	0.002	3.998,1.718-9.229	0.001	3.877,1.666-9.021	0.002	3.297,1.414-7.686	0.006
ΔSUV (< 9.4 vs ≥ 9.4)	1.372,0.649-2.899	0.407	1.575,0.745-3.332	0.235	1.721,0.812-3.646	0.156	1.366,0.646-2.889	0.414
ΔSUV/SUV1 (< 0.68 vs ≥ 0.68)	0.652,0.308-1.381	0.264	0.714,0.337-1.511	0.378	0.767,0.363-1.623	0.488	0.807,0.377-1.726	0.580
Multivariate analyses								
KPS (< 90 vs ≥ 90)	0.409,0.188-0.893	0.025	0.419,0.189-0.929	0.032	0.413,0.187-0.912	0.029	0.291,0.130-0.651	0.003
SUV1 (< 14.2 vs ≥ 14.2)	2.304,0.994-5.337	0.052	2.576,1.098-6.041	0.030	2.620,1.115-6.158	0.027	2.347,1.006-5.474	0.048
SUV2 (< 3.6 vs ≥ 3.6)	2.947,1.213-7.161	0.017	2.892,1.195-7.001	0.019	2.792,1.155-6.750	0.023	2.620,1.083-6.342	0.033

*Abbreviations:* LRFS = local recurrence free survival; DMFS = distant metastasis free survival; PFS = progress free survival; OS = overall survival; HR = hazard ration; CI = confidence interval; KPS = Karnofsky Performance Status; SCC = squamous cell carcinoma; Adeno = adenocarcinoma; GTV = gross tumor volume; MLD = mean lung dose; SUV = standardized uptake values.

For the 22 patients who received induction chemotherapy, Cox proportional hazards regression analyses revealed that neither SUV1 nor SUV2 was associated with LRFS, DMFS, PFS, or OS (*p* > 0.30). For the 62 patients who underwent proton therapy with concurrent chemotherapy only, SUV1 and SUV2 were associated with DMFS, PFS, and OS (*p* < 0.05). SUV2 also predicted LRFS (*p* = 0.037), which is consistent with the analysis of all patients as a group.

## Discussion

This study confirmed our prior findings that concurrent chemotherapy and high-dose proton therapy yielded local control rates in excess of 80% at 2 years and a median OS time of about 29 months for patients with inoperable stage III NSCLC [5]. DM remains the dominant pattern of failure at about 40%. Nodal recurrence outside the PTV (i.e., elective nodal failure) occurred in <10% of patients, but isolated elective nodal failure in only 2%. Our findings suggest that further escalation of the biological effective dose, perhaps as hypofractionated radiotherapy, may be needed in some cases to improve local control; they further suggest that more effective chemotherapy will be crucial for reducing DM. The question at this time is how one might select patients for further dose escalation beyond 74 Gy or for more potent chemotherapy [3]. Our finding that an SUV2 in excess of 3.6 could predict LRFS (*p* = 0.017) suggests that PET/CT imaging during or toward the end of treatment could be helpful for deciding whether additional dose escalation is needed and would allow adequate time to design a boost treatment without requiring a break in therapy.

Our finding that KPS predicted survival was hardly surprising. Interestingly, however, both SUV1 and SUV2 were independent prognostic factors for DMFS, PFS, and OS (*p* < 0.05). A high SUV1 could be an indicator for higher dose or novel chemotherapy, given that a high SUV1 predicted DMFS and OS. This finding is important, because SUV1 was obtained before the treatment began.

Previously, FDG accumulation before preoperative chemoradiotherapy was not associated with pathologic outcome, but FDG uptake by residual tumor masses 2 weeks after induction chemoradiotherapy predicted pathologic response with 88% sensitivity when an SUV cutoff of 3.0 was used; specificity was only 67% because of treatment-related inflammation [18]. This finding is consistent with our own. However, the predictive value of SUV1 remains uncertain. ^18^ F-FDG SUV in the primary tumor before chemoradiotherapy has been shown to predict local-regional failure in NSCLC and a high SUV value within the target volume to correlate with local recurrence [12,19,20]. However, the radiation dose in those studies was <70 Gy (median, 63 Gy). In the present study, all patients received 74 Gy(RBE) proton therapy with chemotherapy, which may have affected the predictive value of SUV1 before therapy. The *p* value for SUV1 and LRFS (*p* = 0.052) was sufficiently close that inclusion of additional patients may demonstrate statistical significance; nevertheless, SUV1 did predict disease progression and DM. Perhaps high-dose proton therapy with concurrent chemotherapy kills most of the cancer cells in the target volume but leaves viable, resistant residual cells that may eventually grow and lead to recurrence. We speculate that a higher PET SUV2 value may indicate the regrowth of such cancer cells. Also, close review of image patterns with respect to the radiotherapy field can be crucial for distinguishing local recurrence from radiation-induced inflammation. Although biopsy can be used to confirm local-regional recurrence, serial images are often obtained as a less-invasive alternative. Prospective studies are needed to clarify the value of SUV2 for predicting LRFS, particularly SUV measurements obtained during or toward the end of proton therapy.

We further found that SUV before and after induction chemotherapy did not predict LRFS, DMFS, PFS, or OS. This finding could result from the small number of such patients in our study. However, SUV2 still predicted LRFS, and SUV1 and SUV2 were still associated with DMFS, PFS, and OS in the patients who received concurrent chemotherapy and proton therapy without induction chemotherapy.

We did not find an association between ΔSUV or ΔSUV/SUV1 and survival, although others have shown such an association among patients with stage III/IV NSCLC treated with chemotherapy [21,22]. Our study was limited by its retrospective nature and the relatively broad interval between completion of therapy and obtainment of the second set of PET/CT images (median 4.2 months, range 0.8–6.0 months). Prospective studies with PET/CT scans obtained at set times, particularly during treatment, are needed to validate our observations and guide further dose escalation.

## Conclusions

High-dose PT with concurrent chemotherapy produced a local control rate of 83.3% on 2 year and median survival duration of 29.9 months in inoperable stage III NSCLC. SUV2 predicts LRFS, and both SUV1 and SUV2 predict DMFS, PFS, and OS. Because of the limited number of patients, the results of the present study need to be further validated in future studies.

## Competing interests

The authors declare that they have no competing interests in this study.

## Authors’ contributions

JYC and ZLX designed the study and performed the statistical analysis and drafted the manuscript. JYC, ZLX, JE participated in acquisition of data. RK and JDC helped study design and analysis. All authors read and approved the final manuscript.

## References

[B1] Curran WJ Paulus R Langer CJ Komaki R Lee JS Hauser S Movsas B Wasserman T Rosenthal SA Gore E Sequential vs. concurrent chemoradiation for stage III non-small cell lung cancer: randomized phase III trial RTOG 9410 J Natl Cancer Inst 2011 103 1452 1460 10.1093/jnci/djr325 21903745 PMC3186782

[B2] Bradley JD Bae K Graham MV Byhardt R Govindan R Fowler J Purdy JA Michalski JM Gore E Choy H Primary analysis of the phase II component of a phase I/II dose intensification study using three-dimensional conformal radiation therapy and concurrent chemotherapy for patients with inoperable non-small-cell lung cancer: RTOG 0117 J Clin Oncol 2010 28 2475 2480 10.1200/JCO.2009.27.1205 20368547 PMC2881726

[B3] Chang JY Zhang X Wang X Kang Y Riley B Bilton S Mohan R Komaki R Cox JD Significant reduction of normal tissue dose by proton radiotherapy compared with three-dimensional conformal or intensity-modulated radiation therapy in stage I or stage III non-small-cell lung cancer Int J Radiat Oncol Biol Phys 2006 65 1087 1096 10.1016/j.ijrobp.2006.01.052 16682145

[B4] Zhang X Li Y Pan X Xiaoqiang L Mohan R Komaki R Cox JD Chang JY Intensity-modulated proton therapy reduces the dose to normal tissue compared with intensity-modulated radiation therapy or passive scattering proton therapy and enables individualized radical radiotherapy for extensive stage IIIB non-small-cell lung cancer: a virtual clinical study Int J Radiat Oncol Biol Phys 2010 77 357 366 10.1016/j.ijrobp.2009.04.028 19660879 PMC2868090

[B5] Chang JY Komaki R Lu C Wen HY Allen PK Tsao A Gillin M Mohan R Cox JD Phase 2 study of high-dose proton therapy with concurrent chemotherapy for unresectable stage III nonsmall cell lung cancer Cancer 2011 Epub ahead of print 10.1002/cncr.26080 PMC3174272 21437893

[B6] Pieterman RM van Putten JW Meuzelaar JJ Mooyaart EL Vaalburg W Koëter GH Fidler V Pruim J Groen HJ Preoperative staging of non-small-cell lung cancer with positron-emission tomography N Engl J Med 2000 343 254 261 10.1056/NEJM200007273430404 10911007

[B7] Lardinois D Weder W Hany TF Kamel EM Korom S Seifert B von Schulthess GK Steinert HC Staging of non-small-cell lung cancer with integrated positron-emission tomography and computed tomography N Engl J Med 2003 348 2500 2507 10.1056/NEJMoa022136 12815135

[B8] Everitt S Herschtal A Callahan J Plumridge N Ball D Kron T Schneider-Kolsky M Binns D Hicks RJ MacManus M High rates of tumor growth and disease progression detected on serial pretreatment fluorodeoxyglucose-positron emission tomography/computed tomography scans in radical radiotherapy candidates with nonsmall cell lung cancer Cancer 2010 116 5030 5037 10.1002/cncr.25392 20623786

[B9] Sulman EP Komaki R Klopp AH Cox JD Chang JY Exclusion of elective nodal irradiation is associated with minimal elective nodal failure in non-small cell lung cancer Radiat Oncol 2009 30 4 5 10.1186/1748-717X-4-5 PMC2651897 19183471

[B10] Fleckenstein J Hellwig D Kremp S Grgic A Gröschel A Kirsch CM Nestle U Rübe C F-18-FDG-PET confined radiotherapy of locally advanced NSCLC with concomitant chemotherapy: results of the PET-PLAN pilot trial Int J Radiat Oncol Biol Phys 2011 81 4 e283 e289 10.1016/j.ijrobp.2011.01.020 21470782

[B11] Weber WA Petersen V Schmidt B Tyndale-Hines L Link T Peschel C Schwaiger M Positron emission tomography in non-small-cell lung cancer: prediction of response to chemotherapy by quantitative assessment of glucose use J Clin Oncol 2003 21 2651 2657 10.1200/JCO.2003.12.004 12860940

[B12] Sasaki R Komaki R Macapinlac H Erasmus J Allen P Forster K Putnam JB Herbst RS Moran CA Podoloff DA [18 F]fluorodeoxyglucose uptake by positron emission tomography predicts outcome of non-small-cell lung cancer J Clin Oncol 2005 23 1136 1143 10.1200/JCO.2005.06.129 15718309

[B13] Mac Manus MP Hicks RJ Matthews JP McKenzie A Rischin D Salminen EK Ball DL Positron emission tomography is superior to computed tomography scanning for response-assessment after radical radiotherapy or chemoradiotherapy in patients with non-small-cell lung cancer J Clin Oncol 2003 21 1285 1292 10.1200/JCO.2003.07.054 12663716

[B14] Hicks RJ Mac Manus MP Matthews JP Hogg A Binns D Rischin D Ball DL Peters LJ Early FDG-PET imaging after radical radiotherapy for non-small-cell lung cancer: inflammatory changes in normal tissues correlate with tumor response and do not confound therapeutic response evaluation Int J Radiat Oncol Biol Phys 2004 60 412 418 10.1016/j.ijrobp.2004.03.036 15380574

[B15] Chang JY Dong L Liu H Starkschall G Balter P Mohan R Liao Z Cox JD Komaki R Image-guided radiation therapy for non-small cell lung cancer J Thorac Oncol 2008 3 177 186 10.1097/JTO.0b013e3181622bdd 18303441

[B16] Moyers MF Miller DW Bush DA Slater JD Methodologies and tools for proton beam design for lung tumors Int J Radiat Oncol Biol Phys 2001 49 1429 1438 10.1016/S0360-3016(00)01555-8 11286851

[B17] Kang Y Zhang X Chang JY Wang H Wei X Liao Z Komaki R Cox JD Balter PA Liu H 4D Proton treatment planning strategy for mobile lung tumors Int J Radiat Oncol Biol Phys 2007 67 906 914 10.1016/j.ijrobp.2006.10.045 17293240

[B18] Ryu JS Choi NC Fischman AJ Lynch TJ Mathisen DJ FDG-PET in staging and restaging non-small cell lung cancer after neoadjuvant chemoradiotherapy: correlation with histopathology Lung Cancer 2002 35 179 187 10.1016/S0169-5002(01)00332-4 11804691

[B19] Abramyuk A Tokalov S Zöphel K Koch A Szluha Lazanyi K Gillham C Herrmann T Abolmaali N Is pre-therapeutical FDG-PET/CT capable to detect high risk tumor subvolumes responsible for local failure in non-small cell lung cancer? Radiother Oncol 2009 91 399 404 10.1016/j.radonc.2009.01.003 19168248

[B20] Klopp AH Chang JY Tucker SL Sulman EP Balter PA Liu HH Bucci MK Macapinlac HA Komaki R Cox JD Intrathoracic patterns of failure for non-small-cell lung cancer with positron-emission tomography/computed tomography-defined target delineation Int J Radiat Oncol Biol Phys 2007 69 1409 1416 10.1016/j.ijrobp.2007.05.085 17904303

[B21] Kim YS Lee MK Kim SJ Kim IJ Kim YK Jo WS Park SK Prognostic stratification using F-18 FDG PET/CT in patients with advanced stage (stage III and IV) non-small cell lung cancer Neoplasma 2010 57 241 246 10.4149/neo_2010_03_241 20353275

[B22] Dooms C Verbeken E Stroobants S Nackaerts K De Leyn P Vansteenkiste J Prognostic stratification of stage IIIAN2 non-small-cell lung cancer after induction chemotherapy: a model based on the combination of morphometric-pathologic response in mediastinal nodes and primary tumor response on serial 18-fluoro-2-deoxy-glucose positron emission tomography J Clin Oncol 2008 26 1128 1134 10.1200/JCO.2007.13.9550 18309948

